# Identifying State Resources and Support Programs on E-Government Websites for Persons with Intellectual and Developmental Disabilities

**DOI:** 10.1155/2015/127638

**Published:** 2015-04-09

**Authors:** Kathleen M. Fisher, Justin D. Peterson, Jon D. Albert

**Affiliations:** ^1^College of Nursing and Health Professions, Drexel University, Mail Stop 501, 245 N. 15th Street, Philadelphia, PA 19102, USA; ^2^Cooper Medical School of Rowan University, 401 South Broadway, Camden, NJ 08103, USA; ^3^School of Medicine, George Washington University, 2300 Eye Street NW, Washington, DC 20037, USA

## Abstract

This descriptive cross-sectional study identified resources and programs that are available nationwide on the Internet to support individuals and families with intellectual and developmental disabilities (I/DD), with a focus on intellectual disability. This evaluation included easily identifiable information on specific resources and highlighted unique programs found in individual states that were linked from e-government websites. Researchers documented the ease of access and available information for all 50 states and the District of Columbia. A number of disparities and areas for improvement were recorded for states and I/DD websites. The researchers conclude that a number of additional health and support services will be needed to address the growing needs of this vulnerable population.

## 1. Introduction

Intellectual and developmental disabilities are lifelong chronic conditions, with multiple etiologies. Further, intellectual and developmental disabilities (I/DD) can affect anyone, that is, from any ethnic or socioeconomic background. They are characterized by limitations in both intellectual functioning and adaptive skills, including learning, language, self-care, and capacity for independent living [1]. Prevalence rates for I/DD vary from 1.5% to 2.5% of the US population [2, 3]. This means that one in ten families, or roughly 30 million Americans, will at some point in their lifetime be directly affected by a person with an I/DD [4].

Unfortunately individuals with I/DD are at increased risk of certain chronic health conditions including seizures, mental health issues, obesity, heart disease, diabetes, osteoarthritis, osteoporosis, vision abnormalities, and poor oral health [5–7]. They often experience disparities in health care including opportunities for health screening and health promotion. If health promotion programs exist, they often are not targeted to meet the specific needs of this population [8]. In December of 2011 those with I/DD were designated a “medically underserved population” by the American Medical Association [9]. Therefore, finding a medical home is difficult and a critical need when individuals “age out” of pediatric practices, and there is evidence that many are followed well into their adult lives by pediatrician and pediatric specialists [10].

Educational experiences can be challenging and limited as well, and opportunities for employment upon completion of primary and/or secondary schooling (if successful) are also limited. Employment opportunities are so limited that it is rare that one with I/DD would earn enough money to be self-sufficient [10]. For example, in academic year 2007-2008, only 34% of students with intellectual disabilities graduated from high school with a regular diploma. Sadly, even with their diplomas, those with intellectual disability were among the lowest paid of graduates with disabilities 1 to 4 years after graduating high school [11]. All of these challenges contribute to significant health and social disadvantages when compared to the general population and highlight the complex planning and coordination needs at both the individual and system level to promote optimal health and quality of life [12]. Consequently, the majority of individuals with I/DD will need varied and individualized lifelong support and services in the communities where they live.

With an increasing number of Americans turning to the Internet for general and health information, we saw a need to evaluate this resource for persons with I/DD. This study was undertaken utilizing the lens of the individual family caregiver to assess and compare programs identified on e-government websites as resources for persons with I/DD. Once the needs for housing and health care have been ensured, a focus on quality of life and socialization becomes paramount for the individual and the family. Another focus of this study was to identify unique services in individual states that could serve as a benchmark for other states.

## 2. Materials and Methods

A descriptive, cross-sectional assessment to identify state services and available programs for the adult with I/DD was conducted by three researchers. Our team collectively assessed all fifty states and the District of Columbia on the use of their respective government-run websites (.gov or  .us) and program links found on their respective pages to communicate to the public which services are offered by the state for adults with I/DD. Next we assembled data by state, and each researcher explored 17 states using the questions identified in the list below. The team met for peer review and presented and assembled lists of resources as a group. Frequency and descriptive statistics were applied to the generated lists. Data collection and analysis was accomplished in the summer of 2011, with additional assessments of targeted e-government websites completed in the fall/winter of 2012-2013.

### 2.1. Procedure

We used the widely known search engine, Google, and a combination of simple search times (i.e., [State] intellectual disability resources and I/DD programs in [State]). Unique features, services, and difficulties encountered were noted for each state. Websites were assessed based on predetermined criteria and questions (see the following).

Criteria used to analyze each e-government website were as follows.

1. Does the website offer state-supported respite care?
2. Does the website offer state-supported transportation services?
3. Does the website offer state-supported residential services?
4. Does the website have specific services for the transition period into adulthood?
5. Does the website recognize the aging I/DD population and offer unique services targeted towards the special needs of this group?
6. Does the state offer assistance in purchasing or renting assistive technologies and medical equipment?
7. What difficulties, if any, were encountered?
8. What unique features or programs did the website offer?
9. Was any contact information listed? If so, what type? (i.e., phone number and email.)

### 2.2. Ethical Guidelines

Information accessed was in the public domain and all sources are cited. Findings presented in this paper are the sole responsibility of the researchers.

## 3. Results

**Figure 1:**
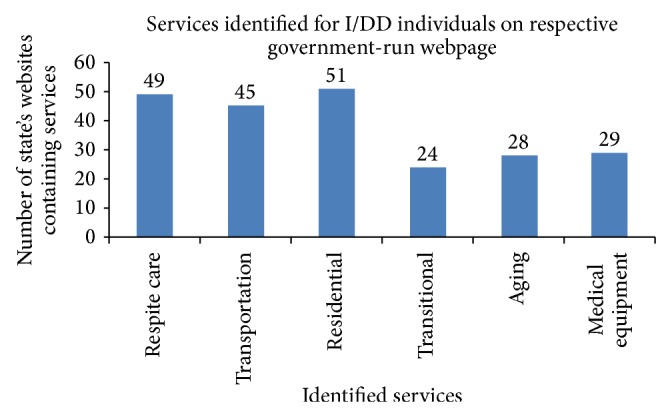
State services for those with I/DD.

Our focus of e-government sites was deemed as appropriate websites for state services and provided uniform comparison among states. Available information on services and ease of access were recorded nationwide. Specifically, available information on services was as follows: residential 100% (51/51), respite 96% (49/51), transportation 88% (45/51), medical equipment 57% (29/51), aging 55% (28/51), and transitional 47% (24/51).  Figure 1 represents the total number of e-government sites (out of 51 evaluated) that had easily identifiable information on specific types of disability service.

For contact information, a phone number was listed 96% (49/51), a phone number with a local area code 65% (33/51), an email 45% (23/51), and an inquiry form 6% (3/51), while only 45% included an email address. Our researchers randomly selected 8 states to email for more information. Four states replied within two days (Arizona, Delaware, Illinois, and Iowa), while the other 4 states never replied to a request for more information on disability services (Alabama, Arkansas, Alaska, and Connecticut). Unique programs identified and the states they are found in include The Wyoming Institutes for Disability (WIND) Assistive Technology Resources (Wyoming) [13], Meaningful Day Initiative (New Mexico) [14], Clyde's Karate (West Virginia) [15], Vanderbilt Kennedy Center Next Steps (Tennessee) [16], Parent Empowerment Network (West Virginia) [17], and Green Mountain Self Advocates (Vermont) [18]; each is described next.

### 3.1. Unique Programs

In our search, we came across dozens of unique programs on different e-government sites, many of which would benefit implementation nationwide or internationally. A few of these programs are highlighted here, offering a template for states to decrease the nationwide disparities in meaningful care. We selected four that demonstrate distinguishing services that enhance the quality of life for those with I/DD. The programs included for discussion were also selected for their innovation and ability to serve as models for programs in other states.

Wyoming, for example, offers the intellectually disabled population one rather intriguing and unique service. On the state's website we found a link to a program called the “Wyoming Institute for Disabilities (WIND) Assistive Technology Resources” [13], which acts as an intermediary for the exchange of medical assistive technologies. The program helps locate used adaptive equipment, which is often donated or loaned through the program. Loans for equipment can last years and are often done cheaply or free of charge. The site operates on a “pay-it-forward” type of platform, which allows users to sign up and both exchange their old medical equipment and locate new equipment they may need. The site currently has over 1,500 items listed, which range from products like walkers to car modifications to electronic devices.

The New Mexico state-run website for developmental disabilities had a subpage dedicated to the “Meaningful Day” initiative [14]. This initiative encapsulates many of the self-autonomous goals of individuals with I/DD. The program promotes freedom of choice, allowing the individual to play a crucial role in the support process. “Meaningful Day” is intended to help build relationships, provide resources for challenging employment, ensure housing in a safe, suitable environment, assist in accessing health care, promote engaging activities, and improve quality of life. Furthermore, this initiative intends to prevent or limit isolation and isolating activities, replacing them instead with engaging activities that would be used by the general population as well. The program has specific guidelines for the retired and elderly individual with I/DD and reflects the importance of an evolving lifelong individualized program of care. This goal is consistent for all individuals with I/DD, regardless of whether they are using waivered state funds [14].

The West Virginia Developmental Disability Council funded Clyde's Karate to provide recreational services, and they developed an instructional DVD of their martial arts classroom [15]. The DVD realistically and honestly profiles the added benefits realized for individuals, their peers, and their parents, as well as the instructor of the class. For example,

 
*one student of this program, Sean, a young man who has Down Syndrome, has had many gains. Sean lost 20 pounds and his coordination, strength, breathing, and endurance improved. Most importantly, he increased his self-discipline, self-esteem, respect, and brotherhood and camaraderie with peers both with and without developmental disabilities [15].*

In Tennessee, a task force to develop a pilot program in postsecondary education for individuals with I/DD was formed in 2007. The “Next Steps” at Vanderbilt University began admitting students in January 2010. The following year, the “FUTURE” program at the University of Tennessee opened its doors for students with I/DD who wished to further their education beyond high school. Both programs are two years in length and award certificates through individualized and person centered planning programs. Both programs share the same goal of increasing employment opportunities for their graduates [18]. The US Department of Education in 2012 awarded funding to 27 institutions of higher education to either create or expand high quality transition and postsecondary programs for individuals with intellectual disability [19].

Our team took notice of numerous recurring problems the average proxy caregiver or person with I/DD might face during their search for state resources. Common problems encountered while searching for available resources included the fact that certain links led to unavailable pages, invalid or nonactive phone numbers and email addresses, no “search” feature, and/or search features that did not provide relevant results. Quite often, state-run websites contained “dead links,” or links that when clicked did not access a valid webpage. Two states were noted for their convenient and helpful search options: Idaho and Kansas. While a few states had definitions for terms like intellectual disability, on rare occasions the words “mental retardation” still appeared in place of the more current and accepted term “intellectual disability.”

Finding contact information for a representative was often challenging, and in certain instances, our researchers could not find an email or phone number for a representative to contact specifically on I/DD services; for example, 98% of states had a phone number listed, while only 45% included an email address. Of the 8 states chosen at random for an email inquiry, only 4 responded. Furthermore, after attempting to contact a number of representatives from various states, we found inactive phone numbers and emails. All of these barriers pose a potential source of frustration for individuals seeking more information on services for persons with I/DD, as they may have a difficult time finding reliable information, a representative to contact, and a response, depending on the state's website they are researching and contacting.

Aging-specific services, for example, appeared in 47% of state websites. In addition to aging services, Aging Disability Resource Centers (ADRCs) [1], links were found on a number of state websites and we suggest that all e-government I/DD sites should provide links to their local ADRCs (if applicable). Knowledge of age-specific services is critically important to our seniors with disability to successfully “age in place.”

Quality of life concerns and the need to establish networks were obvious on a number of websites that included programs (e.g., “best buddies”) to address the isolation and lack of socialization that can often accompany this developmental disability. For many families, a circle of friends beyond relatives is nonexistent for their loved ones with I/DD [10]. The need for connection by both caregiver and individual was apparent and is identified in the number of programs emerging to connect people, both nationally and internationally. The Parent Empowerment Network (PEN), for example, in West Virginia, serves to establish links with carers [16]. Green Mountain Self in Vermont is operated and run by people with intellectual disability. “How to give a speech” and “how to educate yourself” are two important programs on this website that link and socialize individuals [17].

## 4. Discussion

Widespread use of the Internet has led an increasing number of Americans to access medical information online. Reported findings range vastly [20–22], but the data suggests that up to 80% of citizens have turned to the Internet for information on their health or for the health of their loved ones, which is roughly the percentage of adults that access the Internet [23]. Half of the health-related Internet inquiries for health-related issues are on behalf of someone else [20].

Concerns have been raised over the quality of information and accessibility of consumer health information on the Internet, as well as the implications of its increasingly prevalent use for health-related inquiries [21]. With the expanding reliance on the Internet for information, numerous researchers have conducted studies evaluating website characteristics such as reliability, usability, veracity, and accessibility [24–26].

While several studies have had a focus on domestic US websites, some studies have evaluated Internet sources from an international perspective [25–28]. One study developed a website evaluation questionnaire for nursing websites that was tested in both the USA and Taiwan [25], while Newby and Groom [27] evaluated the usability of a UK rehabilitative site and noted room for improvement, specifically in relation to legibility, layout, writing style, and the need for more information. Another study assessed the quality and readability of Internet information for adults with hearing impairment and their caregivers in five different countries and offered a number of suggestions for improvement based on the disparities they found [28]. The literature suggests that readability, reliability, and ease of access are among the commonly found issues in accessing reliable health information online both in the United States and in other countries.

While nearly all states have at least one Aging and Disability Resource Center (ADRC), these centers do not specifically focus on I/DD [29]. All US states have a department or division that provides I/DD services, but there are numerous discrepancies between each state. Consequently, we need to focus attention on resources that provide both formal and informal services that can support a quality of life to “age in place,” as individuals with I/DD are aging and will outlive family caregivers, creating uncertainty for future care giving, health care, and housing needs. Families are struggling to find adequate lifelong care and services, especially those that enhance or support quality of life needs [10, 30].

The National Committee on Quality Assurance (NCQA) in the United States annually analyzes the performance and service of individual states' Medicare and Medicaid health programs [31]. Using over 100 data measures, their findings ranked all fifty states and the District of Columbia from best to worst, identifying both changes in performance and a model for other states to implement improved delivery of service [13, 14]. While access and availability to services differs across state lines, current literature offers very little insight into the actual experience of identifying and accessing developmental disability services. Our approach was to consider the caregiver, prior to the actual utilization of services with a goal of analyzing the ease or difficulty in finding or identifying available resources for individuals with I/DD. This approach was considered important, as decision making for care and services is commonly done by proxy, that is, by family members or caregivers [15, 32].

Website domains signify the level of restriction, if any, one faces when registering for a webpage. Any individual can apply for a  .com or  .org domain name, and while these links may be helpful, the user needs to be aware that reliability and authenticity of these sites can vary. Unlike general domains, domains that have restrictions on who can register for them include “.edu” (reserved mainly for US-affiliated institutions of higher learning), “.us” (reserved mainly for US state and local governments), and “.gov” (restricted to only US government entities). One study notes the following: “Given the multitude of federal laws and policies, one might expect a relatively uniform and high level of accessibility to state Web sites. The empirical evidence suggests otherwise” [33].

Furthermore, research has indicated that individuals with a disability may encounter even greater difficulties in accessing information via e-government websites, as there exists a gap between policy and practice, noting that policy alone is insufficient in adequate action and implementation by state governments [33]. While the internet is not immune to limitations and inadequacies, research suggests it can be a promising source and effective platform for health information, communication, and education [22], and it is an empowering way to put more control in the hands of the public [34].

In our findings, we note that our inability to locate specific services in a state does not conclude nor should it imply that the state does not offer these services. Rather, it can portray the difficulties a caregiver or individual with disabilities may experience in their own efforts to locate state services and resources. In reality, we believe that a higher percentage of states offer the services we screened for. Our experience highlighted that there are numerous disparities across e-government websites and that further research should be conducted to highlight specific difficulties caregivers perceive in their efforts to access services for individuals with I/DD.

*Limitations.* Although our analysis of websites was uniform and our team used the same combination search terms for all states, it is possible that information was overlooked for any number of the states. We attribute any information overlooked as typical of the challenges an average caregiver or person with I/DD may experience in identifying resources and services. Further, the scope of our analysis did not include any criteria to determine whether the websites evaluated were “disability-friendly” and easily navigable by an individual with I/DD.

Websites are always changing, and sites may contain out of date or inaccurate information. Resources, even if identified, are not always properly funded or available, and thus, identification is simply the first step of several necessary to benefit from state services. While this study focused solely on e-government sites and the links contained in them, we do contend that caregivers may not distinguish between the government-run website and non-e-government websites. Nongovernment sites were only mentioned if they had been linked on e-government sites. This narrow focus is a limitation in the fact that this study's findings may not be applicable to the panoply of private (nongovernmental) or local government sites that can offer a wide array of I/DD services.

## 5. Conclusions

The Internet plays a significant role in our society and increasingly Americans are using the Internet to identify health services and programs and for health information. We highlighted some unique programs that could serve as models for other states to enhance quality of life for those with I/DD. Our study suggests that finding information on specific I/DD support services through state websites might prove to be difficult, as availability of information was variable across the United States. Further, this study reveals an appreciable gap in contact information and response from state representatives in regard to inquiring for further I/DD resource information.

The convenience of services offered by an individual state can be diminished by limitations in that state's ability to relay reliable information through their Internet webpages to potential recipients in need of such services. We affirm the call for increased readability, reliability, and presentation of information on health-related websites, specifically for I/DD resource websites in the United States.
